# Serious Adverse Drug Reactions and Safety Signals in Children: A Nationwide Database Study

**DOI:** 10.3389/fphar.2020.00964

**Published:** 2020-08-06

**Authors:** Jean Mendes de Lucena Vieira, Guacira Corrêa de Matos, Fabrício Alves Barbosa da Silva, Louise E. Bracken, Matthew Peak, Elisangela da Costa Lima

**Affiliations:** ^1^ Pharmacy School, Rio de Janeiro Federal University, Rio de Janeiro, Brazil; ^2^ Scientific Computing Program, Oswaldo Cruz Foundation (Fiocruz), Rio de Janeiro, Brazil; ^3^ Paediatric Medicines Research Unit, Alder Hey Children's NHS Foundation Trust, Liverpool, United Kingdom

**Keywords:** serious adverse drug reactions, children, spontaneous reports, off label drug use, safety signals

## Abstract

Children are more exposed to inappropriate medicine use and its consequent harms. Spontaneous reporting of suspected Serious Adverse Drug Reactions (SADR) increases knowledge and prevention of pharmacotherapy risk. Disproportionality measures are useful to quantify unexpected safety issues associated with a given drug-event pair (signals of disproportionality). This cross-sectional study aimed to assess SADR reporting and safety signals for Brazilian children from 0-12 years old, notified between January 2008 and December 2013 from the Brazilian Surveillance Agency (Notivisa). Information from serious reports (gender and age of the patient, event description, suspected drug) was included. Disproportionality analysis based on Reporting Odds Ratios with a confidence interval of 95% was conducted to identify possible signals of disproportionate reporting (SDR). Almost 30% of 1,977 suspected SADR was related to babies (0-1-year-old). 69% of reports happened with intravenous dosage forms, and 35% of suspected SADR involved off label use according to age. Laronidase, miglustat, imipenem/cilastatin, and clofarabine were involved in six or more suspected deaths among 75 deaths reported. There were 107 SDRs, of which 16 events (15%) were not described in the product labels. There was a relatively higher number of SADRs in Brazilian children compared with studies from other countries. SDRs found, (especially drug-event pairs ‘imipenen/cilastatin–pneumonia’ and ‘laronidase–respiratory insufficiency’) should be investigated more. The reports of SADR with IV dosage forms and OL drug use suggest the need for drug research and the use of better dosage forms for children in Brazil.

## Introduction 

Ensuring safety, efficacy, and quality of medicines for the pediatric population is a challenge because data on many medicines are scarce due to the lack of clinical trials involving children (Joseph et al., 2015). As a result, children may be exposed to age-inappropriate medicines that can lead to increased incidence of Adverse Drug Reactions (ADRs) (Bellis et al., 2014). Risk factors associated with ADRs in children include polypharmacy, age, off-label (OL) use, and unlicensed (UL) use (Bellis et al., 2014; Lombardi et al., 2018).

Reports of ADRs screening from surveillance systems may offer information about a different drug‐event combination (or pairs) by the mining of a large volume of data (Harpaz et al., 2012). Such safety signals—a potentially causal association or a new aspect of a known association between an intervention and a set of related events—may be critical for drug regulation and improvements in child health (CIOMS, 2010; Osokogu et al., 2016).

Electronic data use for children’s safety is increasing, especially in North America and Europe (Black et al., 2015). ADR prevalence may vary due to differences in pharmacovigilance and polypharmacy management among low and middle-income countries (Olsson et al., 2015; Angamo et al., 2016). In Brazil, the first exclusive investigation about children from the Brazilian database (*Notivisa*) assessed 3,330 spontaneous reports of suspected ADRs and showed approximately 60% were classified as serious events (Lima et al., 2019). This new study aimed to analyze deaths and other serious ADR reports and to identify safety signals in children.

## Materials and Methods

We carried out a cross-sectional study of children with suspected SADRs notified on the *Notivisa*, created in 2008. *Notivisa* is a computerized system developed by the National Health Surveillance Agency (*Anvisa*) to receive notifications of incidents, adverse events, and technical complaints related to the use of health products and medicines by manufacturers, users, health professionals, and health services—mainly those within the Sentinel Network observatory.

The Sentinel Network is an observatory constituting around 200 (general and specialized) accredited hospitals and other health services for monitoring and reporting adverse drug events. Sentinel functions include strategies for surveillance of ADRs and precise mechanisms for identification and investigation, dissemination of results, mandatory risk minimization plans and integration of other institutions for network hospitals. Training for this network was carried out by Anvisa, and it considers the elements described above, including the seriousness and causality assessment of ADRs. SADRs are investigated in health services by patient safety teams before being reported to *Notivisa* (Brazil, 2011; Brazil, 2015; Teixeira et al., 2017; Mota et al., 2018; Brazil, 2019a).

*Anvisa* supplied data on *Notivisa* website reports as a Microsoft Excel^®^ file for the first years of reporting system consolidation, and only complete data between 2008 to 2013 were available for analysis.

Data on suspected ADRs in children (0-12 years old) were identified and selected by age information on the date of the adverse event or the calculation of the difference between the date of onset event and the patient’s birth date. There were no linkage processes with other databases.

The events were described according to the WHO Adverse Reaction Terminology (WHO-ART) and classified following the International Conference on Harmonisation guidance (CIOMS, 1987; ICH Expert Working Group, 2003; WHO, 2018) on the *Notivisa* database as serious outcomes (i) death; life-threatening; hospitalization; disability (significant or persistent); congenital anomaly, medically important events, and (ii) not serious. All reports classified as ‘not serious’ were excluded.

Information about all suspected drugs reported, including pharmaceutical form and administration route, were collected and encoded using the Anatomic Therapeutic Chemical (ATC) classification system (1^st^ and 5^th^ level). OL use in children was verified from the Brazilian labels (summary of product characteristics) of each drug (approved by Anvisa). Drugs not approved for use by the children’s age group were classified as OL use, and drugs not licensed in Brazil were classified as UL (Gonçalves and Heineck, 2016; Aronson and Ferner, 2017). Age group classification was based on the pediatric stage of development (AAP, 2019).

Disproportionality analysis of ADR reporting was used to identify signals of disproportionate reporting (SDR). SDR refers to statistical associations between drugs and adverse events. The disproportionality analysis method recommended by the European Medicines Agency, namely Reporting Odds Ratio (ROR) and thresholds, is based on its 95% confidence interval and the number of individual cases (European Medicines Agency, 2016). ROR measure is defined by the formula [(a.d)/(c.b)], based on a two-dimensional contingency table where value “a” indicates the number of individual cases that list the target drug P and the target ADR R; value “b” indicates the number of individual cases that list the target drug P but not the target ADR R; value “c” indicates the number of individual cases that list the target ADR R but not the target drug P, and value “d” indicates the number of individual cases that do not list the target ADR R or the target drug P. They should be distinguished from signals that can originate from individual case analysis and epidemiological studies. This method assumes that when a signal (involving a particular adverse event) is identified for a drug, this adverse event is reported relatively more frequently in association with this drug than with other drugs (European Medicines Agency, 2006).

We adopted the following criteria to define a SDR (European Medicines Agency, 2016): number of individual cases is greater than or equal to 3 for active substances contained in medicinal products included in an additional monitoring list defined by the EMA; number of individual cases is greater than or equal to 5 for the other active substances and event belongs to the important medical event terms list. ROR calculations were performed for all drugs with a ≥ 3. Nevertheless, we differentiate between the cases where 3 ≤ a < 5 and a ≥ 5. A situation occurs when c=0, or when all database reports containing a target ADR are associated with only one drug. In this case, ROR cannot be computed. ROR value is arbitrarily set at 99.9 to reflect the presence of a possible SDR.

Other analyses were conducted using SPSS version 22.0 for Windows (IBM Corporation, USA). The chi-square test with a significance level of 5% was used to assess the association between the seriousness of ADR, age group, and the number of drugs prescribed as OL.

The use of *Notivisa* data was formally authorized by *Anvisa*. This study was approved by the Hospital Universitário Clementino Fraga Filho Ethics Committee (registration number 931.400).

## Results

### General Findings

One-thousand-nine-hundred-and-seventy-seven SADRs were assessed, and almost 30% involved 0-1-year-olds. The frequency of serious events was significantly different between pediatric age groups (p < 0.05) (**Table 1**). SADRs were more common in boys (54%) than in girls. However, 1% of the total reports of suspected SADRs did not include a specification of gender. More than 70% of reports were done from hospitals, mainly from the Sentinel Network, by patient safety teams. Approximately 28% of suspected SADRs were associated with death, were life-threatening, and caused prolonged hospitalization (**Table 1**).

**Table 1:** Distribution of SADR reports (n=1977) by patient age in Brazil between 2008-2013.

Seriousness	Baby (aged 0 – 1 y)	Toddler(aged 1 - 3 y)	Preschool(aged 3 - 5 y)	Primary school (aged 5 - 8 y)	Secondary school (aged 8 - 12 y)	Total (%)
1 – Death	24	14	10	13	14	75 (4,0)
2 - Life threatening	102	36	26	19	33	216 (11,0)
3 - Prolonged hospitalization	65	61	44	28	65	263 (13,0)
4 - Congenital abnormalities	2	1	0	0	0	3 (0,2)
5 - Persistent or significant disability	4	3	9	4	7	27 (1,3)
6 - Medically important events	345	230	192	203	423	1393 (70,5)
**Total**	**542**	**345**	**281**	**267**	**542**	**1977**

SADR, Serious Adverse Drug Reaction; y = year(s). Congenital abnormalities: SADR in children related to medicine use during the mother’s pregnancy. Source: Notivisa/Anvisa. P value < 0,001 (Chi Square test).

Two-thousand-two-hundred-and-twenty-nine drugs were identified as being associated with reports of SADRs (mean: 1.1 drugs by report; range from 1 to 6). Anti-infectives for systemic use (n = 1048; 47%), drugs for the nervous system, including non-steroidal anti-inflammatory drugs, (n=384; 17.2%) and antineoplastic, and immunomodulating agents (n=221; 10%) were most commonly involved in suspected SADRs (**Table 2**).

**Table 2:** Distribution of suspected drugs by ATC classification involved in 1977 reports of SADRs in Brazil between 2008-2013.

Description	Frequency	
	[n]	%
**Drug class (ATC)**	**2229**	100
Alimentary tract and metabolism (A)	153	6,9
Blood and blood forming organs (B)	100	4,5
Cardiovascular system (C)	54	2,4
Dermatological (D)	9	0,4
Genito urinary system and sex hormones (G)	2	0,1
Systemic hormonal preparations (H)	56	2,5
Anti-infectives for systemic use (J)	1048	47,0
Antineoplastic and immunomodulating agents (L)	221	10,0
Musculo-skeletal system (M)	51	2,3
Nervous system (N)	384	17,2
Antiparasitic products, insecticides and repellents (P)	24	1,0
Respiratory system (R)	47	2,1
Sensory organs (S)	43	1,9
Various (V)	37	1,7

ATC, Anatomical Therapeutic Chemical Code; SADR, Serious Adverse Drug Reactions. Source: Notivisa/Anvisa.

Intravenous powder or solutions were the main pharmaceutical forms reported (69%), followed by tablets (9,3%), oral solutions (4,5%), and oral suspensions (3,7%). Administration route and pharmaceutical form was indeterminate in 9,6% of reports.

Twenty-five percent (n=564) of drugs present in suspected SADRs reports involved OL use by age, according to the Brazilian label, and less than 1% (n=11) of cases were classified as UL use. The frequency of OL prescriptions was significantly higher in events reported, such as persistent disability, prolonged hospitalization, and death (p<0.05) (**Table 3**).

**Table 3:** Distribution of suspected drugs (total and OL prescription by age) by seriousness of SADR in children in Brazil between 2008-2013.

Seriousness classification	Number of drugs		
	Total	Prescribed as OL (%)	P value
Death	89	26 (29%)	0.043*
Life threatening	239	50 (21%)	
Prolonged hospitalization	306	89 (29%)	
Congenital abnormalities	3	2 (67%)	
Persistent or significant disability	31	12 (39%)	
Medically important events	1561	385 (25%)	
**Total**	**2229**	**564 (25%)**	

OL, Off-label; SADR, Serious Adverse Drug Reaction. Values in parenthesis are percentages. *Statistically significant at level of P < 0.05 (Chi Square test).

### Deaths

There were 75 deaths involving 89 suspected drugs. The majority of deaths were related to either cardiovascular events (22%), respiratory disorders (19%), or general disorders (23%). Sixty-seven percent of these fatal events were not described on Brazilian labels.

Three ATC groups concentrated the major part of suspected drugs: alimentary tract and metabolism (n=26; 29%), antineoplastic (n=19; 22%), and anti-infectives (n=18; 20%). Miglustat and imipenem/cilastatin showed 88% and 25% of OL age use, respectively. Drug indication, as represented by ICD (International Classification of Diseases) classification, showed the predominance of metabolic disorders and infections. These findings are presented in **Table 4**.

**Table 4:** Description of suspected drugs (from main ATC groups reported) involved in child deaths on Notivisa between 2008-2013.

Suspected Drug	ICD Classification (drug indication)	Total reports by drugs (n)/OL or UL* use (n)	Main events^1^ classified by System Organ Class
**A - Alimentary tract and metabolism**		**26 (12)**	
Alfaglicosidase	Glycogen storage disease	3 (0)	Cardiovascular/Respiratory
Calcium gluconate	Not reported	1 (0)	General disorders
Imiglucerase	Lipid deposit disorders	3 (0)	General disorders/Respiratory
Laronidase	Mucopolysaccharidosis type I	6 (0)	General disorders/Cardiovascular/Respiratory
Metoclopramide	Nausea and vomiting	3 (3)	Blood/Cardiovascular
Miglustat	Not reported	9 (8)	General disorders/Gastro-intestinal system/Neurologic/Respiratory
Ondansetron	Nausea and vomiting	1(1)	Cardiovascular
**J - Anti-infectives for systemic use**		**18 (4)**	
Ampiciline	Not reported	3 (0)	Neurologic
Benzylpenicillin	Not reported	1 (0)	Neurologic
Caspofungin	Localized infections of the skin and subcutaneous tissue	1 (1)	General disorders
Ceftriaxone	Acute upper airway infection	1 (1)	Respiratory
Imipenem/Cilastatin	Complications of heart defects; Bacterial pneumonia; Infections due to other mycobacteria; Other bacterial diseases; Unspecified microorganism pneumonia	8 (2)	Blood/General disorders/Cardiovascular/Endocrine/Gastro-intestinal/Musculo-skeletal system/Neurologic/Respiratory system/Urinary
Imunoglobuline (human)	Not reported	1 (0)	Cardiovascular
Meropenem	Not reported	1 (0)	Neurologic
Metronidazole	Other venous embolism and thrombosis	1 (0)	Blood
Oxacillin	Not reported	1 (0)	Skin
**L - Antineoplastic and immunomodulating agents**		**19 (14)**	
Carboplatin	Not reported	1 (1)	General disorders
Carmustine + Cytarabine + Etoposide + Melphalan	Not reported	4 (4)	Cardiovascular
Clofarabine	Lymphoid leukemia	8 (8)*	Blood/General disorders/Cardiovascular/Respiratory
Ifosfamide	Not reported	2 (0)	Gastro-intestinal
Methotrexate	Not reported	2 (0)	Blood/General disorders/Cardiovascular/Gastro-intestinal system/Neurologic/Respiratory/Skin
Rituximab	Adverse effects of primarily systemic action substances	1 (1)	Endocrine
Vincristine	Lymphoid leukemia	1 (0)	General disorders

ATC, Anatomical Therapeutic Chemical Code; ICD, International Classification of Diseases; OL, Off-label; UL*, Unlicensed use; Not reported, no information about drug indication on database.

Drugs classified in other groups: heparin, bosentan, furosemide, gentamicin, hydrocortisone, methylprednisolone, ibuprofen, metamizole sodium, methadone, alpha poractant, fenoterol, fluticasone and salmeterol, technetium (^99m^tc) ethylenedicysteine, coagulation factor viii, coagulation factor IX, II, VII, and X in combination, were also reported. There were four cases, all involving newborns, related to contamination of solutions (parenteral nutrition and sodium chloride).

### Safety Signals

Using the disproportionality analysis, applying a signal threshold of (N) ≥3, results pointed to 65 SDRs involving mostly anti-infectives for systemic use (34%), antineoplastic agents (15%), nervous system drugs (12%), and alimentary tract and metabolism drugs (11%). For (N) ≥5, 42 signals were involved mostly with anti-infectives for systemic use (55%), nervous system (17%), antineoplastic agents (12%), and sensory organs drugs (12%). From 107 signals of disproportionate reporting, 16 events (15%) were not described on the Brazilian label, (**Table 5**). Two pairs were suspected with deaths: imipenen/cilastatin–pneumonia, and laronidase–respiratory insufficiency.

**Table 5:** Drug-event pairs associated with Signals of Disproportionate Reporting (SDR) not described in drug label (Brazilian label).

Suspected Drug	Event description	ROR	Lower bound 95%	Seriousness of suspected ADRs found	Thresholds of number of cases (n)
Alphaglucosidase	Asthenia	66.77	27.10	Medically important	≥5
Amphotericin B	Hypokalemia	54.24	24.95	Life threatening Prolonged hospitalization Medically important	≥5
Ciclosporin	Nystagmus	47.11	13.70	Medically important events	≥3
	Psychosis	15.30	4.30	Medically important events	≥3
	Blurred vision	212.5	37.38	Life threatening Prolonged hospitalization Persistent or significant disability Medically important	≥3
Imipenem and Cilastatin	Pneumonia	42.80	11.68	Death	≥3
Laronidase	Respiratory insufficiency	29.05	7.53	Death Prolonged hospitalization	≥3
	Papules	15.02	3.70	Medically important	≥3
Metoclopramide	Psychosis	15.30	54.27	Medically important	≥3
Oxacillin	Cough	2.94	1.00	Life threatening Prolonged hospitalization Medically important	≥3
Tropicamide	Apnea	75.95	18.33	Life threatening Medically important events	≥3
	Asthenia	33.67	13.89		≥5
	Bradycardia	33.67	13.89		≥5
	Persistent crying	677.71	82.54		≥5
	Dyspnea	11.11	4.72		≥5
Vancomycin	Petechiae	3.38	1.23	Medically important events	≥5

ROR, Reporting Odds Ratio; ADR, Adverse Drug Reaction.

## Discussion

Our study found a higher frequency of SADRs in children (60%) than is generally reported compared to other pediatric studies, in which rates of SADRs reported in national databases ranged from 0% to 66.7% (Aagaard et al., 2010; Smyth et al., 2012). The relatively high reported frequency of SADRs in children in this study may be due to the high proportion of *Notivisa* data attributed to inpatients. Another hypothesis is that this database does not discriminate the adverse drug reactions caused by medication errors. The Adverse Drug Reactions in Children (ADRIC) program in the UK reported the incidence of ADRs for hospitalized children as 15.9% (Smyth et al., 2014). A retrospective analysis based on 5 years of pharmacovigilance studies (2012-2016) in an Italian hospital observed 834 ADRs, of which 239 (29%) were serious (Lombardi et al., 2018).

One study reported age as a risk factor for an ADR (Lombardi et al., 2018). Our results suggest evidence of an association (p<0.05) between age and the seriousness of an ADR (**Table 1**).

Vancomycin (n=9,2%), Ceftriaxone (5,9%), and Oxacillin (n=5,7%) were the specific drugs most commonly reported as being associated with a suspected SADR in the current study. Anti-infectives and antiepileptics represent the most frequently reported therapeutic classes associated with ADRs in children admitted to hospital, and non-steroidal anti-inflammatory drugs were often reported as associated with ADRs in outpatient children (Smyth et al., 2012). Our findings agree with this review, although the proportion of ADRs associated with antiepileptic drugs was small (1%).

The intravenous route represented the most common dosage form (69%) associated with a suspected SADR, which also may be attributable to the Sentinel Network notifications being dominated by inpatients. However, this route represents a higher risk for the incidence of medical errors than with other formulations (Grissinger, 2010), which reinforces the necessity of discussions about the selection and development of better formulations for children in Brazil. Furthermore, cases of contamination of sterile solutions have been reported, which may be related to problems in preparing and administering these products for newborns.

The Food and Drug Administration (FDA) and EMA implemented regulatory initiatives for pediatric medicines in the early 2000s. Both agencies created legislation to perform studies in children for products seeking marketing authorization in adults that meet certain criteria based on the relevance to public health (Turner et al., 2014). These initiatives have had a positive impact on pediatric drug development, and pediatric considerations have become an integral part of pharmaceutical development across the US and Europe (European Comission, 2016; Food and Drug Administration, 2016).

In Brazil, even with the participation of *Anvisa* in the Pediatric medicines Regulators’ Network (PmRN) (WHO, 2010), no similar initiative was taken by any Brazilian regulatory authority regarding pediatric drug development. Nevertheless, prioritization of license analysis of a new drug, further pharmaceutical form, new therapeutic indication, and new concentrations for children (fast track) can be requested (Brazil, 2017).

Regarding other policies to ensure safer pharmacotherapy, Brazilian regulatory standards for manufacturers currently do not provide differentiated strategies for monitoring drugs used in children. *Anvisa* became a member of the International Council for Harmonization of Technical Requirements for Pharmaceuticals for Human Use (ICH) in 2016. There was a public consultation for update rules (in 2018) in which the Periodic Benefit-Risk Evaluation Report (PBRER) and the pharmacovigilance planning would meet the criteria adopted by ICH, concerning the information on the benefit-risk profile of drugs in special populations, such as children. Unfortunately, the new regulation is not yet published due to challenges in the resources required to complete the guidance *Anvisa* (Brazil, 2019b). Such challenges in the development and dissemination of territory-specific safety guidance can increase the risk associated with the use of children’s medicines in middle-income countries like Brazil.

The percentage of drugs associated with any serious SADR level, which used OL, was 25% (range 21-67%) (**Table 3**). Children are especially vulnerable to SADRs due to the lack of data for this age group and the extensive use of OL and UL drugs (Elzagallaai et al., 2017). Reducing OL use in children could decrease the impact of SADRs, such as prolonged hospitalization, persistent disability, and death, as shown in **Table 3** (p<0.05). In 2013, a prospective cohort study conducted in a pediatric hospital in the United Kingdom evaluating the impact of OL and UL prescribing on ADRs causing admissions to a pediatric hospital showed a 23% increase in ADR risk due to OL use (Bellis et al., 2014).

Of the SADR reports which were associated with deaths, babies (0-1-year-old) (n=24; 32%) were the age group most implicated (**Table 1**). Neoplasms, respiratory, infectious and parasitic, related nervous or cardiovascular system diseases, and chromosomal abnormalities or congenital malformation stand out as causes of mortality and or morbidity in Brazilian children (Vieira et al., 2017). Considering that most records were from hospitalized children, it was expected that serious ADRs in Brazil would involve babies and drugs with related indications, as shown in **Table 4**. Furthermore, drugs indicated for metabolism disorders (ICD class E) and infection diseases (ICD class J), as observed in **Table 4**, are well described in literature such as patterns of off-label prescribing in children (Luedtke and Buck, 2014; Gonçalves et al., 2017). In severe clinical conditions, there is a complex assessment of the balance between risks and benefits, and, even if there is a higher chance of a fatal adverse event, the use of the drug might be the best medical decision. However, labels are a primary source of safety information from manufacturers for healthcare professionals and must be up to date with the frequency of ADRs in order to take precautionary and management measures in case of serious events (Brazil, 2010).

There were only three reports classified as congenital anomalies. Although these are not drugs directly administered to children, damage to the fetus was suspected, making this information relevant for the pharmacotherapy monitoring and evaluation in special populations, including pregnant women. The pediatric disease burden in low-and middle-income countries is particularly affected by congenital anomalies, and the spontaneous notification of these events, as expected, was meager, which reinforces the demand for studies with better power to recognize these cases (WHO, 2020).

The present analysis described a high number of fatal events (67%) and were also associated with signals of disproportionate reporting (15%) (**Table 5**) not expressed in the product label.

Three drugs were suspected of eight or more fatal events: Miglustat, imipenem/cilastatin, and clofarabine. Of the suspected drugs associated with death, there was a high proportion of OL/UL use and specifically OK use, and, specifically the OL use (e.g., miglustat and imipenem/cilastatin) and UL use (e.g., clofarabine) associated with them (**Table 4**).

Disturbances such as diarrhea and flatulence are the most common adverse effects associated with miglustat therapy (Belmatoug et al., 2011; Remenova et al., 2015. In one fatal case, this event was present (**Table 4**). For imipenem/cilastatin, nausea and vomiting were also reported in the literature (Oliva et al., 2005) and was present in cases of deaths. Most of the events reported in the literature for clofarabine, such as blood disorders (e.g., neutropenia) and respiratory disorders (e.g., respiratory distress), have been described (Jeha et al., 2006; O’Connor et al., 2011) (**Table 4**).

Star et al. analyzed (2019) pediatric individual case safety reports from Vigibase and founded 27 potential signals. After an in-depth assessment, they pointed out eight signals involving dextromethorphan (OL use), olanzapine (accidental overdose), atomoxetine (two signals), temozolomide, deferasirox, levetiracetam, and desloratadine. They used a data-driven predictive model that prioritized reporting a series of emergent signals by weighing disproportionate reporting patterns, completeness, recency, and geographic spread of individual case reporting and availability of case narratives.

Although it is one of Anvisa’s goals, the Notivisa data analyzed in this study could not be sent to the Uppsala Monitoring Center, due to the incompatibility of notification systems, this study suggests the identification of new signals. However, we acknowledge that local practices, specific characteristics of the pediatric population, and pharmaceutical characteristics used in Brazil may have influenced our results.

We found 16 pairs involving serious events that need more assessment and follow-up. Imipenem/cilastatin and laronidase must be monitored for the risk of pneumonia and respiratory insufficiency, respectively. These pairs were reported as fatal events. A case report of imipenem/cilastatin-induced acute eosinophilic pneumonia in a 60-year-old woman (Foong et al., 2016) reinforces a suspected link of this ADR.

Disproportionality analysis in pharmacovigilance databases is an exploratory and reliable method to generate signals. Once a new disproportionality ratio for a drug has been observed, further studies can be conducted to confirm the signal (Montastruc et al., 2011).

Spontaneous reporting is considered an accessible and low-cost pharmacovigilance method that provides drug use assessment in real-life situations for large sample sizes (Pal et al., 2013). However, underreporting of adverse reactions makes it impossible to estimate the actual frequency of events. Another limitation of secondary database use is missing data, which is particularly important for causality assessment. Although notifiers were trained for a prior analysis of the suspected ADR, some of the necessary data had not been captured by *Anvisa* and cannot be undertaken retrospectively.

## Conclusion

We found substantial SADRs reporting from the Brazilian database involving IV dosage forms and OL drug use in children. Imipenem and cilastatin, laronidase, clofarabine (unlicensed use), and miglustat (off label use) were suspected drugs related to deaths which were more frequently reported in the study period.

Moreover, the high number of signals of disproportionate reporting observed, mainly for anti-infectives, suggests that the safety profile of these medicines in children should be further investigated.

Finally, we recommend the dissemination and use of information from the periodic safety update reports for children, the development of pediatric formulations, and specific policy approval in Brazil following the examples of pediatric drug regulations in the US and Europe.

## Data Availability Statement

The datasets generated for this study are available on request to the corresponding author.

## Ethics Statement

The studies involving human participants were reviewed and approved by Hospital Universitário Clementino Fraga Filho Ethics Committee. Written informed consent from the participants’ legal guardian/next of kin was not required to participate in this study in accordance with the national legislation and the institutional requirements.

## Author Contributions

EL conceptualized the study. JV and EL led data collection, carried out the analysis, and drafted the initial manuscript. GM, FS, LB, and MP assisted with interpretation of the data and preparation of the final version of the manuscript. All authors contributed to the article and approved the submitted version.

## Funding

This study was funded by the National Council for Scientific and Technological Development (CNPq) (Grant number: 421992/2016-6).

## Conflict of Interest

The authors declare that the research was conducted in the absence of any commercial or financial relationships that could be construed as a potential conflict of interest.
